# Spontaneous Transanal Small Bowel Evisceration with Distinct CT Findings: A Case Report

**DOI:** 10.2174/0115734056410969251111150236

**Published:** 2025-11-28

**Authors:** Young Min Song, Sung Hwan Bae, Sung Woo Jang

**Affiliations:** 1 Department of General Surgery, Soonchunhyang University Seoul Hospital, Soonchunhyang University College of Medicine, Seoul, Korea; 2 Department of Radiology, Soonchunhyang University Seoul Hospital, Soonchunhyang University College of Medicine, Seoul, Korea

**Keywords:** Transanal evisceration, Rectal perforation, Computed tomography, Small bowel loops, Hartmann procedure

## Abstract

**Introduction:**

Transanal small bowel evisceration is an extremely rare and life-threatening surgical emergency that primarily occurs in debilitated elderly patients. Preoperative computed tomography (CT) can be useful for identifying the viability of eviscerated small bowel and other intra-abdominal pathologies.

**Case Presentation:**

In this study, we report the case of an 81-year-old woman who presented with sudden anal protrusion of small bowel loops. Computed tomography (CT) demonstrated a rectal wall defect, pneumoperitoneum, and herniation of the small bowel with features suggestive of strangulation. Emergency laparotomy revealed a firmly impacted ileal segment plugging a perforation at the rectosigmoid junction, likely due to increased intra-abdominal pressure, necessitating small bowel resection and the Hartmann procedure. Early diagnosis and prompt surgical intervention led to a favorable postoperative course.

**Conclusion:**

This case highlights the critical role of CT in identifying rectal perforation and intrarectal small bowel evisceration.

## 1 INTRODUCTION

Transanal small bowel evisceration (TSBE) is an extremely rare and potentially life-threatening condition requiring emergency surgical intervention. It may occur spontaneously or after rectal perforation, likely due to blunt trauma [1-5]. In spontaneous cases, rectal prolapse has been frequently reported as a predisposing factor [5-7]. Rectal perforation can lead to panperitonitis, whereas an eviscerated small bowel carries a high risk of strangulation, emphasizing the need for prompt diagnosis and urgent surgical management [4, 6, 8].

Although preoperative computed tomography (CT) can be useful for identifying small bowel ischemia and evaluating other intra-abdominal pathologies, detailed descriptions of CT findings in previously reported cases of transanal evisceration are limited [9]. Here, we present a comprehensive diagnostic and surgical approach, focusing on unique CT findings, for an elderly patient with TSBE.

## 2 CASE REPORT

**Fig. (1):**
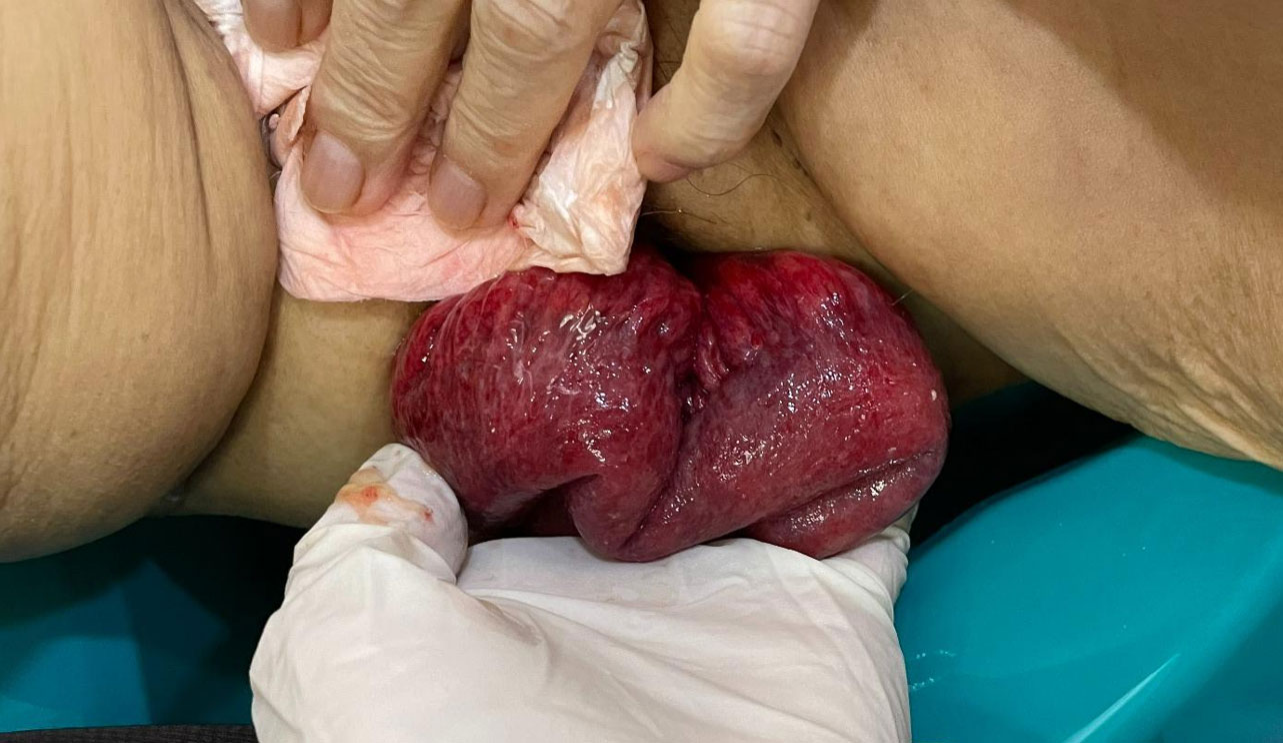
Small bowel evisceration through the anus.

An 81-year-old woman presented to the emergency department with a lump protruding through the anus that had developed on the same day (Fig. **1**). The patient was alert with mild cognitive impairment. The patient’s initial vital signs included a blood pressure of 112/76 mmHg, a pulse rate of 106 beats/min, a respiratory rate of 16 breaths/min, and a body temperature of 37.4°C.

A closer examination revealed that the protruding mass was in the small intestine along the mesentery. Manual reduction of the prolapsed bowel was unsuccessful, and the patient reported a persistent urge to defecate. The patient did not complain of abdominal pain. On physical examination, the abdomen was soft, with no tenderness or rebound tenderness. Laboratory results showed leukocytosis with a white blood cell count of 15,300/µL and an elevated C-reactive protein level of 9.45 mg/dL. Despite relatively stable vital signs and unremarkable findings on abdominal examination, the presence of a small bowel protruding through the anus raised a strong suspicion of bowel ischemia, necessitating urgent surgical intervention.

**Fig. (2):**
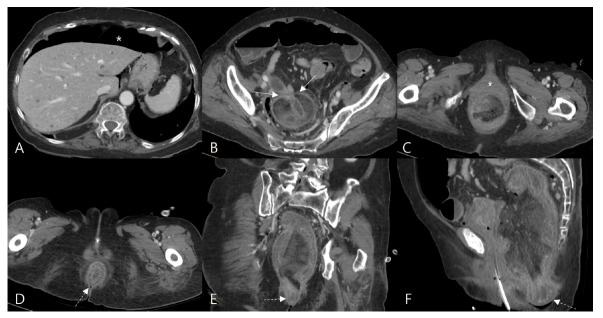
Pre- and post-contrast-enhanced abdominopelvic CT images in an 81-year-old woman. Axial contrast-enhanced CT images (**A**-**D**), coronal reformatted image (**E**) and sagittal reformatted image (**F**). Axial image (**A**) shows a large amount of intraperitoneal free air (white asterisk). Axial image (**B**) demonstrates a large wall defect (white arrows) in the rectum, with small bowel loops and mesentery herniating into the rectal lumen. Axial image (**C**) shows herniated small bowel and mesentery in the anal canal. Axial, coronal and sagittal images (**D**-**F**) reveal small bowel loops (white dashed arrows) protruding externally beyond the anal verge, suggestive of transanal evisceration. The herniated bowel loops exhibit diffuse edematous wall thickening with decreased bowel wall enhancement, highly suspicious of strangulation. **Abbrevation:** CT = computed tomography.

Preoperative contrast-enhanced abdominopelvic computed tomography (APCT) was performed for further evaluation. APCT revealed a large amount of intraperitoneal free air suggestive of bowel perforation (Fig. **2A**). A significant defect in the rectal wall was identified with small bowel loops and mesenteric herniation into the rectal lumen (Fig. **2B**). The distended rectum was occupied by the herniated bowel, with some loops extending through the anal canal and protruding externally (Fig. **2C**-**F**). The affected small-bowel loops showed diffuse wall thickening, edema, and decreased enhancement, suggesting strangulation and ischemia (Fig. **2B**-**F**).

The patient had a history of dementia and subdural hematoma. For the past two months, she had been spending most of her time in bed because of back pain from a compression fracture in the second lumbar vertebra. She experienced symptoms of rectal prolapse one month earlier but refused surgical intervention at that time. Additionally, her husband reported that she tightly fastened an abdominal binder throughout the day to alleviate the back pain, which possibly led to increased intra-abdominal pressure. The patient’s body mass index was 21.5 kg/m^2^ (height, 155 cm; weight, 51.7 kg).

**Fig. (3):**
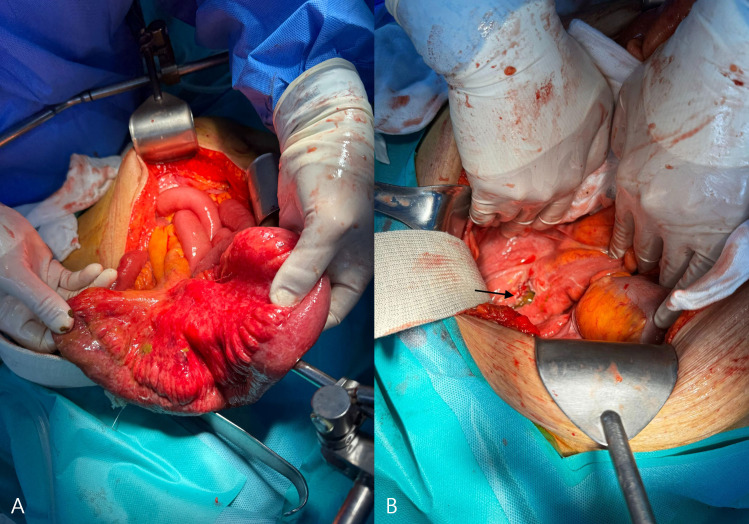
Operative findings. (**A**) Ischemic changes in the eviscerated small bowel. (**B**) Perforated rectal wall (black arrow).

During the exploratory laparotomy, a segment of the ileum was firmly impacted by a perforation at the rectosigmoid junction. An 80 cm segment of ileum, located 30-110 cm from the ileocecal valve, was ischemic and required resection (Fig. **3**). After manual reduction of the herniated bowel, the rectal wall defect was found to be approximately 4-5cm in length with poorly defined and friable margins. Due to the extent of the tear and compromised tissue viability, a proctosigmoidectomy with end colostomy (Hartmann operation) and segmental resection of the strangulated small bowel were performed. Notably, intra-abdominal contamination was minimal, likely because the impacted small bowel sealed the rectal perforation, limiting peritoneal spillage. This finding may account for the absence of significant abdominal pain or peritoneal signs on physical examination.

Postoperatively, the patient was kept nil per os, intravenous crystalloids, and antibiotics. The patient’s postoperative course was uneventful, and she was transferred to the general ward three days after dietary initiation. The patient was discharged on postoperative day 6 with stable colostomy function and bowel recovery.

## 3 DISCUSSION

First reported in 1827 [1], TSBE is an extremely rare and life-threatening condition, with < 100 cases documented in the literature [2-4]. It typically occurs in elderly or debilitated patients with underlying predisposing factors, such as chronic rectal prolapse, increased intra-abdominal pressure, or connective tissue weakness [3, 5, 6]. Other risk factors include chronic constipation, previous pelvic surgery, trauma, and direct injury [2, 7, 8].

Rectal prolapse plays a critical role in the pathogenesis of the disease. Chronically prolapsed rectal tissue is subjected to repeated shear stress and progressive ischemic changes, leading to thinning and weakening of the rectal wall [10, 11]. Shear stress refers to the mechanical stress exerted tangentially on tissues and can compromise microvascular perfusion, resulting in localized ischemia and structural vulnerability [12]. Consequently, the weakened segment becomes prone to perforation, particularly when subjected to sudden increases in intra-abdominal pressure. Once a perforation occurs, the small bowel loops may initially herniate through the rectal wall (transrectal evisceration) and prolapse externally through the anal canal, resulting in transanal evisceration.

In this case, longstanding rectal prolapse and the use of an abdominal binder to manage back pain likely contributed to the increased intra-abdominal pressure, resulting in rectal wall perforation and subsequent small bowel evisceration.

Contrast-enhanced CT is useful for diagnosing rectal wall defects, identifying herniated bowel loops within the rectal lumen, detecting pneumoperitoneum, and evaluating bowel viability. In this case, contrast-enhanced APCT clearly visualized the rectal perforation and transanal herniation of the small bowel loops, which have rarely been documented previously [13-15]. Although CT findings are valuable for diagnosis, they may not always accurately assess the viability of the herniated bowel, necessitating clinical judgment and intraoperative assessment.

Notably, patients with transanal evisceration may not exhibit the classical signs of peritonitis despite rectal perforation. This phenomenon can be attributed to the eviscerated small bowel itself sealing the perforation site, thereby limiting fecal contamination and preventing the rapid onset of panperitonitis [1, 15]. Consequently, the initial clinical presentation may be mild. However, as the ischemic injury progresses in the herniated small bowel, the patient’s condition rapidly deteriorates to sepsis. Therefore, appropriate diagnosis and timely surgical intervention are critical to prevent severe complications.

Outcomes are generally favorable if prompt diagnosis and timely surgical management are achieved [2, 5, 8]. Although small bowel ischemia can progress to septic conditions, the process is slower than that of fecal peritonitis, providing a therapeutic window for successful intervention. Surgical strategies vary depending on the intraoperative findings, patient condition, and surgeon preference. Although laparoscopic reduction may be considered in selected cases, it is often not feasible in this clinical context. In our case, the herniated small bowel was severely impacted, and laparoscopic instruments might have caused further bowel injury and contamination due to spillage of bowel contents. Therefore, Hartmann’s surgery is commonly performed in elderly or unstable patients. Primary repair could be considered; however, primary repair of the rectal defect is generally not recommended in unprepared bowel conditions due to the risk of poor healing and leakage. Small bowel resection was indicated if ischemic changes were evident in the herniated segment. Considering that this condition predominantly affects elderly patients with potential comorbidities, meticulous postoperative care is critical to optimize outcomes and minimize complications.

## 4 STUDY LIMITATIONS

Due to its nature as a single case report, the findings are not readily generalizable to other clinical scenarios. Due to the patient's cognitive impairment, a detailed medical history or subjective experience could not be reliably obtained. Additionally, the emergent clinical setting limited the opportunity for a comprehensive evaluation of potential contributing factors, such as underlying rectal pathology or pelvic floor dysfunction. Further case series or systematic studies are needed to clarify the underlying mechanisms and guide optimal management strategies for spontaneous transanal small bowel evisceration.

## 5 PATIENT’S PERSPECTIVE

As the patient had cognitive impairment, she was unable to fully understand the nature of the event or the treatment she received. However, she expressed general satisfaction during her recovery, stating that she felt comfortable and was eating well. Her daughter, who remained at her bedside throughout the hospitalization, confirmed that the patient appeared content and thankful for the medical care provided.

## CONCLUSION

TSBE is a rare but potentially fatal condition requiring prompt diagnosis and urgent surgical intervention. Preoperative CT is a valuable tool for identifying rectal defects and assessing the associated bowel injuries, although clinical judgment is essential. Therefore, clinicians must maintain clinical awareness and ensure timely surgical intervention to prevent severe complications, such as strangulation, ischemia, and sepsis.

## Data Availability

The data and supportive information are available within the article.

## LIST OF ABBREVIATIONS

TSBE: Transanal Small Bowel Evisceration
CT: Computed Tomography
APCT: Abdominopelvic Computed Tomography

## STANDARDS OF REPORTING

CARE guidelines were followed.
